# Can COVID‐19 mitigation measures promote telework practices?

**DOI:** 10.1111/wusa.12498

**Published:** 2020-11-18

**Authors:** Ammar Abulibdeh

**Affiliations:** ^1^ Department of Humanities, College of Arts and Sciences Qatar University Doha Qatar

## Abstract

The propagation of coronavirus disease 2019 (COVID‐19) pandemic had resulted in a significant slowdown of economic activities worldwide. Teleworking practices and policies could play a significant and essential role of any business continuity plan in time of risk that prohibits employees from performing their working tasks at their regular workplace or offices. Hence, telework gives those employees the opportunity to perform their work tasks remotely and keep the organization operational. The aim of this study is to examine the impact of the COVID‐19 pandemic on promoting telework practices. This study discusses the transition to telework during the COVID‐19 pandemic and the continuity of teleworking practices after the pandemic. Furthermore, the study discusses what elements and interventions that are necessary to maximize the potential gains for more widespread telework for employers and employees. Adopting telework in this circumstance can reduce the negative impact of the pandemic and be a mitigating measure for the economy and inequality. This research is important because it analyses the benefits of implementing telework practices in a time of risk.

## 1 INTRODUCTION

Coronavirus disease 2019 (COVID‐19) is a novel strain of coronavirus first identified in 2019 in Wuhan, the capital of Hubei, China. The disease has spread globally, resulting in the 2019–2020 coronavirus pandemic. This virus is similar to other coronaviruses such as Severe Acute Respiratory Syndrome (SARS) and Ebola and has infected millions of people and caused hundreds of thousands of fatalities worldwide. The health crises translated to an economic crisis as the governments started to take measures to curb the spread of the pandemic. Countries took two fundamental strategies in dealing with the pandemic. First strategy focused on mitigating the adverse effects of the pandemic, while the second relies on more stringent measures to suppress and reverse the growth trajectories (Cowling & Aiello, 2020; Depellegrin, Bastianini, Fadini, & Menegon, 2020; Nikhat & Fazil, 2020; Qi et al., 2020; Tobías, 2020). These strategies were adopted and implemented by many countries across the globe particularly the developed countries.

Governments have implemented containment measures to mitigate the negative affect of the pandemic on the public health as well as to limit and stop the spread of COVID‐19 and hence to “flatten the curve” to reduce the number of new infected cases related to the disease and to sustain and promote public welfare. These measures include financial, monetary, and fiscal policy measures (Gourinchas, 2020), such as preventing or limiting travelers' entry; canceling thousands of flights; closing borders partially or fully; major nationwide lockdowns for weeks; imposing a 14‐day self‐quarantine for nationals returning from affected countries; closing schools, colleges, universities, community centers, and nongovernmental organization; suspending public transportation partially or fully; prohibiting mass gatherings; closing most businesses; and urging people to work from home. In addition, governments have encouraged or forced residents to remain in their homes to limit their contact with others and encouraged them to practice social/physical distancing (Ali & Alharbi, 2020; Bonaccorsi et al., 2020; Nicola et al., 2020). Under these circumstances, people require permission to perform their essential excursions such as for food and medicine (Weder di Mauro, 2020). However, these policies vary between countries in scope and breadth.

Implementing these containment measures affected “essential” and “nonessential” industries differently and resulted in dramatic lay off and high unemployment, budget deficits, disruption in supply chain, shutdown of financial markets, high inflation rate, high poverty rate in the majority of economies, and billions of people across the globe have self‐quarantined in their homes. The consequences result in declining trade activities, global demand, and investments (Campello, Kankanhalli, & Muthukrishnan, 2020; Forsythe, Kahn, & Lange, 2020). Therefore, there is a risk of another global recession that will be worse than the one occurred in 2008.

Governments announced supportive packages to support and protect their economies from the negative impacts and against the harmful damage of the pandemic. Some developed countries, such as the United States, Canada, the United Kingdom, Japan, Italy, and Spain have implemented historical stimulus measures to manage the economic damage from the pandemic. Simultaneously, some developing countries, such as China, Indonesia, Qatar, Oman, and South Africa, have adopted substantial policy incentives to support their economies. Furthermore, international organizations, such as the United Nations, the International Monetary Fund, the World Bank, the Organization for Economic Co‐operation and Development (OECD), and the European Central Bank, initiated certain actions to support countries and human lives, as well as to stabilize the global economy. These actions include fiscal and monetary measures. Such resolutions have aimed at mitigating the adverse impact of the virus on the economy; however, the magnitude and length of the economic downturn depends on several elements, such as the size and duration of the pandemic, the effectiveness of the stimulus packages in supporting business and individual sentiments, and the extent of containment measures and economic lockdown. These measures will have negative consequences on economic growth, both directly and indirectly. The developing countries who are already suffering from a weak economic situation will have considerable problems in bearing the economic costs from the spread of COVID‐19.

The economic impacts of COVID‐19 were unpredictable, unprecedented, and inflict the economic activities in the majority of countries worldwide and are not limited to the number of countries that were severely infected by the disease. Global economy is expected to shrink up to 4.9% during the second quarter of 2020 (Brodeur, Clark, Fleche, & Powdthavee, 2020; International Monetary Fund, 2020) and will be losing $9 trillion due to the pandemic (Tahir & Batool, 2020). Rubini (2020) provided a theoretical discussion of a paradigm shift and economic interventionism needed as a result of the pandemic to transform the way societal production is conceptualized due to the demise of markets and capitalist systems. This paradigm shift must deprioritize economic growth and must furnish an alternative by the political economy. The starting point is by balancing between needs and wants, and in creating an environment that would achieve a sustainable and equitable increase in societal well‐being. He suggested that the COVID‐19 pandemic must be considered a market failure and that it has the potential to create a three‐layer economic crisis, starting at a point of production. Furthermore, the economic crisis resulted from the pandemic necessitates a shift from the profit‐centered neoliberal paradigm toward the society focused on sustainable, efficient, and equitable development. The impact on the economy, individuals' income and their ways of life, and the digital revolution in recent decades is a motivation for rethinking telework as a fundamental option to support the economy. The digital revolution has delivered immense opportunities and transformed the way of life and societies at an unprecedented scale and speed. However, although technology is crucial for telework, technology per se is insufficient for a successful telework process. On the national scale, telework should be assimilated into the business strategy and supported by government policies and good management. On the international scale, telework requires international collaboration, especially regarding technology. These conditions are necessary to achieve the full social and economic potential of telework and avoid negative externalities on the national and global scales.

The aim of this study is to provide an analysis of the impact of COVID‐19 pandemic mitigation measures on promoting telework practices. The study analyzes the impact of the pandemic on the labor market, sectors, and countries and the transition to telework practices. Furthermore, the study discusses the inequalities resulted from the pandemic on the countries and the workers and how teleworking can mitigate these inequalities. This study provides a holistic review of teleworking practices and suggest actionable recommendations for effective teleworking to support policymakers as well as both public and private sectors in developing or updating existing teleworking policies. This study explores the literature from recent months that gives more insight on the importance of teleworking practices in time of risk. Although this study focuses on examining the importance of teleworking practices during a pandemic or emergency situation, which requires an emergency response to ensure business continuity, the study as well can be applied to promote teleworking practices in general outside the scope of risk.

## 2 THE IMPACT OF THE COVID‐19 PANDEMIC ON THE LABOR MARKET, SECTORS, AND OCCUPATION

### 2.1 The impact of COVID‐19 pandemic on the labor market

The impact of the COVID‐19 pandemic on the labor market is of wide ranging, affecting most organizations and workers to different degrees. The mitigation policies imposed by the governments had dire consequences on workers, including furloughs, mandatory leaves of uncertain duration, layoffs and job loss, business closures, and reducing working hours and wages. On the other hand, the pandemic has led to an increase workload for some jobs such as the medicine and biomedical sectors, as well as major changes in working conditions and arrangements including short‐time work and teleworking.

Several studies investigated the impact of COVID‐19 pandemic on the labor market (see, e.g., Adams‐Prassl, Boneva, Golin, & Rauh, 2020; Béland, Brodeur, & Wright, 2020; Coibion, Gorodnichenko, & Weber, 2020; Gupta et al., 2020; Kahn, Lange, & Wiczer, 2020; Rojas et al., 2020). Between March 12 and April 12, the employment rate in the U.S. falls by about 1.7% points for every extra 10 days because of implementing the stay‐at‐home mandate (Gupta et al., 2020). In the United States, job vacancies and job posting have been dramatically declined, while the unemployment rate had increased due to the pandemic associated by an increase in discouraged workers and the coverage by unemployment insurance (UI) had witnessed a steep rise (Coibion et al., 2020; Kahn et al., 2020; Tuman, 2020). The decline in job vacancies was uniform across occupation, industries, and states. Furthermore, the new hiring ads have dropped by 40% below the average level of postings during the first week of May 2020 compared with the same week in 2017–2019 (Campello et al., 2020). High‐ and low‐skill jobs postings in the United States witnessed reduction during the pandemic (Campello et al., 2020). Down skilling and new hiring cuts are more conspicuous in local labor market in areas with greater income inequality, in low‐income areas, in the nontradable sector, in industries where workers are more unionized, and in areas where employment is concentrated within few organizations (Campello et al., 2020). In South Korea, Aum et al. (2020) found that the increase of the infected cases results in reducing local employment even in the absence of implementing government‐mandated lockdowns. The number of unincorporated self‐employed workers fell 12.6% including those reporting being employed but absent (Kalenkoski & Pabilonia, 2020; U.S. Bureau of Labor Statistics, 2020a, 2020b). Another study found that the number of actively working unincorporated self‐employed workers fell 28% (Fairlie, 2020). Kalenkoski and Pabilonia (2020) investigated the early effects of COVID‐19 lockdown on the employment and hours of unincorporated self‐employed workers. The study found that coupled men were more likely to be working than coupled women, while single men is less likely to be working than single women. Moreover, the study found that men without children who remained employed are more likely to work normal hours compared to fathers of school‐age children. Béland et al. (2020) found that the less negatively affected by the pandemic are occupations that have a higher percentage of teleworkers and those who are classified as “more exposed to disease.”

### 2.2 The impact of COVID‐19 pandemic on the sectors

The impact of the COVID‐19 pandemic varies when related to the different sectors in varies degrees. The manufacturing industry has been affected by the propagation of the pandemic, and staffing deficiencies and importation issues have been key concerns for businesses due to self‐isolation policies and the disruption of the supply chain (Kahn et al., 2020; Nicola et al., 2020). In addition, the educational sector has been influenced by the pandemic at all levels of the education process and systems, from preschool to tertiary education. The majority of the countries completely closed schools and universities, and initiated online and distance learning for all students. The aim of these closures is to prevent the propagation of the pandemic within educational institutions and hence to prevent the transmission of the disease to vulnerable individuals. Around 68% of the student enrolled in schools (1.2 billion) are affected by school closures across the globe (Nicola et al., 2020; UN, 2020). The impact of school closure could cost on average $142 per student per week in the U.S. cities as a result of the propagation of the pandemic (Nicola et al., 2020).

Travel and recreational industries, as well as hospitality, have witnessed international distortions (with impact on both travel supply and demand), crucial economic loss, and significant declines in terms of tourist numbers and reservations, and consequently a reduction in the number of workers (Gössling, Scott, & Hall, 2020; Nicola et al., 2020; Tuman, 2020). The pandemic has also affected the travel industry, particularly the aviation sector, which is grappling with the significant drop in demand and the unprecedented wave of cancellations due to border closures and the restrictions on nonessential travel (i.e., tourism travel, immigrant visas, and work visas) imposed by many governments. The UN World Tourism Organization estimated the potential loss of international tourists to range between 850 million and 1.1 billion, with between $910 billion and $1.2 trillion of potential loss in export revenues from tourism, and between 100 and 120 million jobs estimated to be at risk (UN World Tourism Organization [UNWTO], 2020). The International Air Transportation Association announced that the air travel industry would lose $84.3 billion because of the outbreak of COVID‐19, and that 2020 would be the worst year in history for airlines, with the losses continuing into 2021, albeit to a lesser extent. Sports industry and food sectors were also affected by the spread of the virus. Many sport events were suspended, postpone, or canceled such as Euro 2020 tournament, Tokyo 2020 Olympics, national football tournament in Europe and elsewhere, the National Basketball Association (NBA) tournament in the United States, boxing, rugby, tennis, and many others. This will have a crucial economic and financial burden in the short run and long run. Regarding the food sector, including food distribution and retailing, it is affected because of people panic‐buying and stockpiling, which has result in increased concerns in terms of food products availability in the short run. The pandemic also results in food insecurity in many countries and it is estimated that the pandemic will double the number of people facing food crises, particularly from the low‐ and the middle‐income countries.

### 2.3 The impact of COVID‐19 pandemic on the enterprises

From the enterprise's perspective, the pandemic has different negative consequences because of the short‐term closures such as decline in job posting and loss of productive workers. Large and small enterprises in the United States have witnessed decline in active job posting and their hiring has reduced by over 30–50% during the pandemic (Campello et al., 2020). Bartik et al. (2020) found that many small businesses in the United States have temporarily reduced their number of workers and closed shops. These businesses were not optimistic about the efficacy of the fiscal stimulus implemented by the federal government. Campello et al. (2020) found that job losses have been concentrated in and were more severe for industries with highly concentrated labor market, credit constrained firms, and nontradable sectors.

In many developing countries, small‐ and medium‐sized enterprises (SMEs) and the informal workers are the backbone of the economy. SMEs as well as the daily wage earners and migrants are bearing the greatest burden of the pandemic shocks on the enterprise side. Therefore, these countries are considering these sectors when design their stimulus packages. It was estimated that around 60% of the informal workers (1.6 billion) lost their income. Those workers are with no access to social protection and with little or no savings. In the first quarter of 2020, the global working hours declined by an estimated 4.5% compared to the fourth quarter of 2019 (precrisis situation). This is equivalent to around 130 million full‐time jobs (International Labour Office [ILO], 2020a). Compared to the same period, it is expected that the global working hours in the second quarter to be 10.5% lower, which is equivalent to 305 million full‐time jobs. The main reason of this rise between the first and second quarters is the prolongation and extension of containment measures taken by many countries. The percentage of workers living in countries with recommended or required workplace closures has declined from 81 to 68% during April, 2020 driven by the easing and lifting of workplace closures in some countries mainly China (ILO, 2020a). Currently, 81 and 66% of employers and own‐account workers, respectively, live and work in countries affected by recommended or required workplace closures, with significant impact on income and jobs (ILO, 2020a). In lower‐middle‐income countries, employers and own‐account workers are affected as these countries are economies with high level of informality and limited fiscal means and policy space to respond to the needs of such enterprises and own‐account workers.

Many organizations and companies requested their employees to telework full‐time to mitigate the propagation of the pandemic given the health risks associated with performing their work tasks from the workplace. In the short run, some studies found that enterprises witnessed increase in teleworkers productivity (Ozimek, 2020), while other studies reported a decrease in self‐reported worker productivity (Morikawa, 2020). The differential effects of COVID‐19 on enterprises and occupations may determine which policy responses are likely to be effective in reestablishing a well‐functioning labor market. They can inform us about the extent and speed of a potential recovery.

## 3 TELEWORK: DEFINITION, ADVANTAGES, AND LIMITATIONS

For over 40 years, telework (or telecommuting) has been of interest to policymakers, researchers, and practitioners (Groen, Triest, Coers, & Wtenweerde, 2018; Hunton & Strand, 2010; Nilles, 1975). Telework is defined as combining technology, location, and time as a platform for work. This work is performed outside the fixed work location, changing the location‐bound condition of work (Bélanger & Allport, 2008). Telework is a flexible work arrangement that enables employees with the aid of information and communications technology (ICT) to accomplish their work in various locations instead of a fixed, central worksite (de Vries, Tummers, & Bekkers, 2019; Eurofound and ILO, 2017; IOL, 2020b). ICT is a crucial element when transforming work toward greater flexibility, effectiveness, productivity, and responsiveness (Baruch, 2000; Davey, 2012). ICTs provide an advantage to telework practices through diminishing the spatial and temporal boundaries between different work activities, supported multitasking. This advantage helps teleworkers effectively and efficiently coordinate different tasks, speed up work processes, and accomplish projects efficiently (Cardona, Kretschmer, & Strobel, 2013). Notably, the traditional time limitations have lost their significance, and the greater adaptability of work has led to flexible working hours.

Numerous studies have suggested different advantages of telework, for example, improvements in teleworkers' productivity (because of the flexibility in scheduling individual tasks; (Tremblay & Darchen, 2010), work–family balance (Jones, 2015), and job satisfaction (Hill, Miller, Weiner, & Colihan, 1998). Furthermore, the advancement in technology allows teleworkers to be always connected through their personal devices (e.g., smartphones, laptops, tablets) outside their workplace and the time allocated for work activity. At the organizational level, advocates argue that telework has several advantages such as lower overhead costs (e.g., supplies, office space, utilities, parking), improved competitive advantage, development of sustainable buildings, improved facility management, and the creation of organizational efficiencies (Gill, 2006). Telework has significantly changed how individuals and groups interrelate to accomplish work and reach the desired incomes, and how organizations conduct and manage business (Bélanger, Watson‐Manheimb, & Swan, 2012; Pinsonneault & Boisvert, 2001).

On the other hand, different telework limitations were identified in the literature that discourage organizations from adopting and implementing this practice. Despite advancements in ICT, telework creates new complexities in the workplace dynamic for employees. This complexity makes the connectivity of communication, coordination, and management more challenging and induces the work–home conflict (Mahler, 2012). Furthermore, the absence of the best workers when needed, problems using ICT, decreased individual productivity, and a loss of synergy at all levels in the organization are identified among the limitations of implementing telework practices (Pinsonneault & Boisvert, 2001; Watson‐Manheim, & Bélanger, 2002). Other drawbacks of telework are social and professional isolation, contentions and tensions to teleworkers, work intensification, presenteeism, limited collaboration and engagement opportunities, technostress, limited conductivity to teamwork, and health and safety (de Vries et al., 2019; Eurofound and ILO, 2017; Groen et al., 2018; A. S. Smith, Patmos, & Pitts, 2015). Consequently, many organizations have banned or limited to a large extend telework practices (Felstead & Jewson, 2000; Goldstein, 2003). To overcome these limitations, the literature has suggested practices, for example, smart work centers, work hubs, and co‐working centers, to elevate the collaboration between teleworkers. However, in cases such as the situation that has resulted from COVID‐19, these practices are not feasible.

## 4 TELEWORK PRACTICES PRIOR, DURING, AND POST THE COVID‐19 PANDEMIC

### 4.1 The popularity of telework practices across countries, sectors, and occupation prior to the pandemic

Prior to the pandemic, telework practices varied substantially between countries, sectors, and occupation. Within the OECD and the European Union (EU) countries, for example, the extent of people teleworked varied across these countries, from around 25% or less in Portugal, Greece, Poland, Czech Republic, and Italy to around 30% or more in Denmark, Netherlands, and Sweden (Eurofound and ILO, 2017; IOL, 2020b; OECD, 2020). In the United States, some studies indicate that around 20–37% of the workers performed telework practices either regularly or occasionally (Jones, 2015). While, the percentage is significantly less in other countries across the world such as Japan (16%) and Argentina (1.6%) (Eurofound and ILO, 2017).

Teleworking varied across the sectors and was more common in knowledge‐intensive services and least common in manufacturing and less‐intensive service market. These differences reflect task requirements. Many high skilled jobs in knowledge‐intensive industries can be done through teleworking using ICT technological devices such as laptops. On the other hand, physical presence is essential to perform jobs in manufacturing, construction, agriculture, electricity or water supply, mining, and accommodation among others.

Regarding occupation, teleworking was more common among high skilled occupations (e.g., professionals, managers) indicating that many occupations prone to be done remotely for now require high skills. Cognitive and noncognitive skills receive the highest returns in digital‐intensive industries (Grundke, Marcolin, Nguyen, & Squicciarini, 2018). However, continuing digitization may further increase the range of tasks to be done remotely (Autor, 2014). Telework practices were lowest, but relatively frequent, among low‐ and medium‐skilled workers.

In the last few decades, although some countries have encouraged employers to voluntarily apply work flexibility, telework has never been adopted as a policy by any government. Employers can refuse to implement this policy; hence, governance control over telework arrangements is insufficient (Hegewisch, 2009). Many factors may encouraged occupational resistance to working from home. These factors include lack of trust, lack of interest, and desire to invest in the infrastructure required to telework, traditionalism, generational composition of the occupational group.

### 4.2 The transition to telework practices during the COVID‐19 pandemic

Because of the COVID‐19 pandemic, many companies are facing the need to secure business sustainability to maintain jobs and well‐being of their employees and simultaneously they are confronted with the dilemma of safeguarding a healthy and safe environment for their workers. In this emergency circumstances, employers must be ready to adapt and vary their expectations to maintain their businesses and clients. Governments, organizations, and individuals are likely to reshape their perceptions of telework practices because of the economic and social shock resulted from the COVID‐19 pandemic. Some companies may already have a teleworking policy, while others are forced to adopt this policy because of the emergency situation caused by the pandemic. The key to successful implementation of this policy is when employers and employees share responsibilities and join efforts to sustain the business. The ability of many employees to telework has mitigate the resulting economic crisis (Barrero, Bloom, & Davis, 2020; Bick, Blandin, & Mertens, 2020; Brynjolfsson et al., 2020; Montenovo et al., 2020). Therefore, teleworking turned to be a crucial practice for both the private and public sectors in maintaining and sustaining the economy and work activities during the lockdown period of the COVID‐19 pandemic. During the pandemic, sectors, firms, and employees have continued to operate remotely, provided they had the necessary technological, digital, and legal security conditions. This has large impacts on businesses, whether they had embraced teleworking in the past or not (OECD, 2020).

Teleworking has become increasingly prevalent after the enforcement of stay‐at‐home mandates and social distancing measures. The extent to which economic activity is negatively affected by social distancing and stay‐at‐home policies depends mainly on the capacity of organizations to maintain business processes from the homes of the employees (Alipour, Falck, & Schüller, 2020; Papanikolaou & Schmidt, 2020). Brynjolfsson et al. (2020) found that the there is a direct relationship between the number of COVID‐19 infected cases and willingness of employees to switch to work remotely in the United States. However, the study finds that those people working from their homes are more likely to claim UI compared to workers who still commute to their workplace.

In Europe, 40% of the employees started teleworking after the government issued stay‐home order (Eurofound, 2020). However, this percentage varies between the countries in the EU and ranges between 60% in countries such as Finland, 50% in Belgium and the Netherlands, and 40% in Austria and Ireland. Furthermore, around 24% of employees that have never experienced telework before the pandemic start telework during it, compared to 56% of employees, who practice telework occasionally before the pandemic. This indicates that the increase of telework practices can be achieved using the right technology tools and work reorganization. In Japan, less than 13% of employees in the country were able to telework in March 2020 (Dooley, 2020). Employers increased interaction with their employees working remotely due to the COVID‐19 pandemic. Nearly 88% (9 in 10) of employers have increased their communication with their employees regarding health and safety tips. Furthermore, 84% of employers have provided advice on teleworking (WorldatWork, 2020).

Brynjolfsson et al. (2020) conducted a survey in the United States to evaluate the impact of the pandemic on telework practice and found that about 50% of the employees are working from home, including 35.2% indicated that they were commuting pre‐COVID‐19 pandemic and switch to perform their work from home. Furthermore, they found that 10.1% of the employees being furloughed or laid off, and that younger people, employees in information work (i.e., professional, management, and related occupations) were more likely to switch towards working from home. Dingel and Neiman (2020) found that 37% of jobs can be performed from home. These jobs mainly involve face‐to‐face interaction. Avdiu and Nayyar (2020) argued that the supply of labor in industries with home‐based work capabilities and face‐to‐face interactions might be least affected by the pandemic. However, those industries and occupations are more likely to experience negative productivity shocks. Ozimek (2020) conducted a survey among 1,500 hiring managers in the United States and find that 94% of them indicate that some of their employees teleworked during the pandemic. Brynjolfsson et al. (2020) conducted another survey that represented U.S. population and they find that among 25,000 respondents, 34% indicated having switched to telework. Teleworkers are working extra hours and in their free time to meet the demand of work due to the prevailing pandemic (Eurofound, 2020; McCulley, 2020). Reisenwitz (2020) found that employees are spending more time in team check‐ins or one‐on‐one meetings due to physical separation of teams.

Switching to teleworking was not a smooth or a simple transition for all organizations or enterprises, particularly for those with no or limited prior experience with teleworking. Different factors played crucial role for difficult and unsmooth transition to teleworking including the organization characteristics and culture, the lack of the appropriate ICT devices and tools, lack of skilled and trained employees to support the transition to telework practices, data security concerns, and health and safety concerns among others. Furthermore, the job characteristics may play a significant role in performing it entirely through teleworking practices. Prior to the pandemic, many workers in different countries, sectors, and occupations used to perform some of their work tasks remotely; however, these jobs may not be suitable to be done entirely through teleworking. For example, 57.2% of workers in Sweden reported having done some telework in 2015, only 30.7% of current jobs could be reported having done from home (Boeri, Caiumi, & Paccagnella, 2020). The differences between countries and in cross‐country in the teleworking practices might be based on the industrial structure of the countries as well as the nature and characteristics of the occupational tasks, which may more closely reflect constraints to telework.

### 4.3 Teleworking practices post to COVID‐19 era

Some countries decide to reopen the economy and the workplaces in the summer as part of entering the next phase of managing COVID‐19 pandemic. Therefore, employers prepared for the return of their employees to these workplaces. However, the reopening process was not static and it was subject to a second wave of the propagation of the virus as many countries in Europe are experiencing (i.e., Spain, United Kingdom, Italy, and Germany). Furthermore, the uncertainty surrounding the development and deployment of a safe and effective vaccine combined with the lack of an appropriate therapeutic options will endanger and restrain the economic recovery and the return to the normal norm of organization's practices. Therefore, until the vaccine is developed and deployed, governments, enterprises, and employees will benefit from the experience they gain during the last period of the pandemic to adapt to teleworking, which will require new behavior and new norms. These new arrangements will involve teleworking, which incorporates a hybrid or blended form of isolation and deconfinement. A more widespread use of telework can have a wide range of impacts on firm performance and worker well‐being. Policies are key to enable firms and workers alike to benefit from the many opportunities offered by the more common use of telework. This in turn can have positive effects on aggregate productivity and well‐being.

Mass telework practices were initiated after government‐mandated lockdown in an emergency situation in many countries. For many countries, particularly developing or transitional economies, the notion of teleworking is new, while postindustrial economies (United States, United Kingdom, Germany, Nordic countries) witnessed deeply embedded teleworking practices in existing organizations at all levels prior to the pandemic. Going forward, it will be paramount for many governments in the developing and developed world to play a central role in drawing out the lessons learned from the transition to telework practices so far and evaluate its advantages and disadvantages on maintaining the running of the economic activities, and apply these lessons to revise existing or initiate new teleworking policies.

Several studies have found that a very high percentage of employees are willing to telework more often in the future even after physical distancing restrictions will be lifted. Furthermore, many employers have realized that the work tasks can be performed outside of their premises. In addition, many managers who used to resist that their teams perform their work tasks from home are less reluctant to these practices after experiencing that it can be done remotely from home. Likewise, employees have realized that they can perform the work tasks from outside of their traditional office spaces and they become more confident and comfortable using technology in their work (IOL, 2020b). J. Smith (2020) found that nearly 29% SMEs of 1,000 SME in the United Kingdom are planning to increase flexible working postpandemic. Customers and clients are also have been more willing to accept that the services they require are being delivered remotely by teams and employees working from home (IOL, 2020b).

The ongoing COVID‐19 pandemic forced many private enterprises and public sector to introduce teleworking on a large scale. This may lead to adopting this option on a wider scale after the pandemic. Public policies are significant to ensure that new, efficient, and welfare‐improving working methods emerged during the pandemic are maintained and developed once the mitigation measures are over. The expanded use of telework may become part of the job practices and norms for years to come supported by the transformation to knowledge‐based and digital economies. This requires a high degree of inventiveness from governments, stakeholders, social partners, employers, and employees. This new era of teleworking requires the encouragement from the governments by issuing supportive policies. Employers need to consider new kind of management that is based on trust, results based, and take into account the challenges that might encounter the workers particularly family–work balance. This will result in a new way of working that requires to be more flexible, autonomous, and considers more adequately employees circumstances and preferences.

In the future, both public sector and private enterprises employers may consider having a more percentage of their employees' teleworking beyond the pandemic. Furthermore, they need to prepare or update a teleworking and return‐to‐work policy based on their experience they gain during the pandemic. These policies should consider the employers' and the employees' experience regarding what work tasks were performed remotely in a smooth way and what should be improve, and what and how are the jobs have been transitioned to teleworking. Furthermore, these policies should focus on the technological aspects such as the potential need for investment in the new digital technologies necessary to conduct business remotely on the long‐term basis by teleworking more efficiently. COVID‐19 pandemic can promote teleworking policy as a key element and an effective contingency plan in maintaining and sustaining the productivity, job preservation, and business continuity. This goal can be achieved by that both employers and employees share responsibility and commitment. The pandemic may act as a catalyst to promote teleworking practices particularly for public sector with potentially positive spillover effects for productivity also in the market sector. However, teleworking policies must be regularly evaluated and be dynamic to stay relevant to respond to the changing needs in the context of emergency situations.

In the longer run, smarter adoption of efficient telework practices may increase the productivity performance of many firms resulting in raising worker's well‐being and simultaneously lowering firms' costs and could speed up the transition into a “new normal.” In a recent U.S. poll, 61.9% of hiring managers stated their intention to rely more on teleworking in the future (Ozimek, 2020).

As long as the number of infected cases is increasing, telework is significant in well‐being safety and sustain businesses and economic activities. However, the importance of telework might extend to the next phases of the pandemic. Assuming the next phase is when the vaccine or a viable treatment against the virus is available and deployed, still there will be a high degree of uncertainty associated with this phase, which force organizations to take different measures to ensure the safety of their employees. These organizations have to implement strict hygiene and safety regulations such as imposing physical distancing in narrow places such as elevators, meeting rooms, and offices, which result in difficulties for the entire workforce to return safely to the workplace. Therefore, teleworking will remain crucial and important for at least some part of the workforce during this phase.

The full and final impact of COVID‐19 pandemic on labor market yet remains to be determined. Nevertheless, the rate of telework practices in many economies, particularly postindustrial economies, will remain significantly higher than they were before the beginning of the pandemic. The public and private sectors have to plan for future scenarios considering teleworking arrangements to prepare for any future crises, particularly on a short notice to have a smooth transition to teleworking. A number of elements are essential to determine if a job can be performed from home. Factors such as economic and occupational structure, the ability to access to broadband internet, and owning a personal computer are important determinants for a successful telework practices. The amenability of telework increases with the level of economic development of the country (ILO, 2020c). The countries that are advanced in technology and where a large proportion of jobs are in ICT, finance and insurance, teaching, professional services, public administration sectors can encourage and mobilize a greater proportion of the workforce to telework. Other countries that rely heavily on sectors such as agriculture, tourism, manufacturing, and construction are less likely to do so.

## 5 TELEWORK AND INEQUALITIES BETWEEN COUNTRIES AND WORKERS

### 5.1 The inequalities between countries

The main reasons of the inequality in adopting and implementing teleworking between countries are the differences in technology (digital divide) and the economic characteristics (postindustrial, industrial, transitional, or developing) of these countries. In the postindustrial economies, the importance of knowledge, services, and research grow at the expense of manufacturing. Over the past 40 years, many countries have transformed their economies into knowledge‐based economies and digital economies and are characterized now as postindustrial countries such as the United States, United Kingdom, and other European countries. The knowledge‐based economy has had significant implications on telework practices because the investment in knowledge enables teleworkers to be knowledgeable and highly educated workers who can use this knowledge to work from home. Furthermore, incorporating high‐technology intensity, particularly ICT, at the organizational level allows teleworkers to intensively use acquired technology to perform their duties and responsibilities and achieve organizational goals. On the other hand, teleworkers can contribute in the transition to the knowledge‐based economy by using ICT. Working from home by using ICT technologies ensures that the economy will remain running under the quarantine circumstances. The functioning of the economy is becoming increasingly inseparable from the digital economy, digital technology is underpinning ever more transactions, and the velocity of technological change is increasing rapidly. The literature of the past few years has focused on digitalization, which is the transition of businesses through the use of digital services, products, and technologies (Brennen & Kreiss, 2014). The development of the digital economy is associated with the evolving use of internet of things (IoT), advanced robotics, artificial intelligent (AI), big data analytics, and three‐dimensional printing.

The term digital divide represents the inequality in approaching and achieving a digital economy because of economic inequalities among countries. Digital development classifies countries as information rich and information poor. Information poor countries have lower economic development; hence, the adoption of the ICT is low because of the lower rates of educational attainment (Chinn & Fairlie, 2010; Dewan, Ganley, & Kraemer, 2010; Pohjola, 2003). This is a reflection of the ICT and adaptation of telework practices.

Different indicators have been identified in the literature to define the digital divide. These indicators can reflect the opportunities generated by a successful telework practice adaptation in many countries. Among these indicators is the percentage of households with internet access and broadband connections. Obviously, the decrease in internet availability and accessibility negatively affects telework practices because the core of this practice is internet use. Other indicators are, for example, the percentage of e‐government service ability, percentage of population regularly using the internet to find commercial information, number of secure servers per million inhabitants, percentage of population regularly using e‐learning services, and percentage of population using the internet to interact with public authorities (Çilan, Bolat, & Coskun, 2009; Cuervo & Menéndez, 2006). These indicators and how people use ICT allow for the analysis and understanding of the extent that telework practices are feasible in different societies, particularly those in information poor countries.

These indicators allow for investigations into how to narrow the digital divide between communities and enhance digital development in information poor countries; however, this also requires the collaboration of international community to strengthen and enable the environment to promote telework practices by narrowing the gap in digital development, particularly between information rich and information poor countries, developing policies that adopt and enforce regulations that promote telework, and building the capacity of the private and public sectors. In addition, the digital divide gap can be narrowed by focusing on certain technologies because of the cross‐technology diffuse effects of these combined technologies. Because digitalization affects different countries in various manners, the digital divides highlights the collaboration between the international community to form new policies and regulations that lead to a more equitable distribution of digitalization. Therefore, local governments require a policy space to regulate the digital economy and promote and legitimate telework practices. In addition, local governments must narrow the digital divide gap on the national scale among individuals, businesses, organizations, households, genders, incomes, education levels, and geographic areas at different socioeconomic levels, regarding their opportunities for internet access and use.

### 5.2 The inequalities between sectors and workers

The economic crises associated with COVID‐19 pandemic will disproportionately affect enterprises and workers and may interact with the existing inequalities in different dimensions such as gender, age, and ethnicity. Furthermore, differential access to telework practices during the pandemic may exacerbated existing inequalities in the workplace and between enterprises. For example, employees who work in jobs requiring physical presence such as construction cannot practice teleworking. Those employees usually consist from young and less educated workers at the bottom of the wage distribution (Brussevich, Dabla‐Norris, & Khalid, 2020).

Adams‐Prassl et al. (2020) investigated the inequality in income/job losses in the United States and the United Kingdom based on individual characteristics and the type of job. The study finds that those who are not able to perform their tasks from home are more likely to lose their jobs. In terms of income loss, the study finds that individuals that are young and those without a university education and on low income are significantly more likely to experience drops in their income or lose their jobs compared to older and higher‐income workers. Yasenov (2020) found that immigrants, younger adults, and those who are with lower levels of education are less likely to do their tasks from home. Alstadsæter et al. (2020) found that vulnerable population, including parents with younger children, are disproportionately affected by the pandemic in Norway. In the United States, the COVID‐19 pandemic has unequal impact across genders and ethnicity groups. Women and racial minorities such as Latinos and African Americans have been disproportionate and negatively affected (Adams‐Prassl et al., 2020; Alon, Doepke, Olmstead‐Rumsey, & Tertilt, 2020; Forsythe et al., 2020; Yasenov, 2020). Alon et al. (2020) concluded that the closure of schools and day‐care centers forced working women to put more time and efforts toward their children, which would have an adverse impact on working mothers/single mothers. Fairlie, Couch, and Xu (2020) found that Latino and African Americans were disproportionately affected by the pandemic. This is due to that the minority workers mainly work in nonessential services in addition to their lower skills. Borjas and Cassidy (2020) found that the employment rates of immigrant men fall more compared to native men in United States. Bartos, Bauer, Cahlikova, and Chytilová (2020) found that the pandemic magnifies hostility and discrimination against foreigners, particularly from Asia. Kalenkoski and Pabilonia (2020) found differential effects of the lockdown by parental status, couple status, and gender due to the decreased employment and hours for all groups resulted from implementing the lockdown measures to mitigate the spread of the disease. They concluded that the negative effects of the lockdown can be mitigated by teleworking or working in an essential industry. Blundell, Dias, Joyce, and Xu (2020) studied the impact of COVID‐19 pandemic on inequalities and found that younger workers are more worried from the collapsing labor market. Furthermore, self‐employed and low‐income workers have either experienced a drop in economic activity or lost their jobs. Female, lower‐paid, and some ethnic minority workers, who are the core of many jobs, face more health risks as they are more exposed to social contact.

The current pandemic elevated the existing social and economic gender inequalities and challenges for women, which resulted in that companies and enterprises become more gender responsive in their actions related to talking the COVID‐19 pandemic (UN Women, 2020). Women's employment is hit more severely by the pandemic induced economic crises more than men (ILO, 2020c). Women's employment is concentrated in the service‐oriented sectors of the economy, which is classified as nonessential (Alon et al., 2020). Furthermore, due to school and day‐care closure and the increase responsibilities of taking care of the children at home, women, particularly those with young children, are more affected than men and their ability to work outside or inside the home is affected (Montenovo et al., 2020; Zamarro, Perez‐Arce, & Prados, 2020). On the other hand, the gender child‐care gap in the United Kingdom has dropped, as furloughed men increased their participation in household‐provided child‐care (Sevilla & Smith, 2020). Other studies found that 33% of working mothers and 10% of fathers in two‐parent households, respectively, provided all of the care for children while schools where closed in early April in the United States (University of Southern California Center for Economic and Social Research, 2020; Zamarro et al., 2020).

The care‐giving responsibilities of parents have increased given the simultaneous closing of schools and day‐care facilities. In Europe, for example, 36% of workers live in households with children aged under 17 years (Eurofound, 2020). Consequently, this effected self‐employed parents more than self‐employed nonparents. Furthermore, female self‐employed parents may be affected more than male self‐employed parents due to gender norms within the home in addition to gender differences in risk preferences (Kalenkoski & Pabilonia, 2020; Sent & van Staveren, 2019; Sevilla & Smith, 2020). Gender inequality also may be impacted by couple status and parental status (Kalenkoski & Pabilonia, 2020), since women are more likely to enter self‐employment than men to attain flexibility over the timing of their work to balance work and family demands (Abulibdeh, 2018; Gimenez‐Nadal, Molina, & Ortega, 2012). Furthermore, a self‐employed worker's employment may also be impacted by the employment status and industry of a spouse as families juggle household and child‐care responsibilities (Alon et al., 2020).

### 5.3 Telework and opportunities to reduce inequalities

The COVID‐19 pandemic might leave short‐ and long‐term challenging legacies for inequality between countries, enterprises, and workers. Some developing or transitional economies may have challenges to address these inequalities given the levels of debt. On the enterprises level, smaller companies may struggle to survive or announce its bankruptcy, while some big companies may struggle for the short term. However, the pandemic may bring opportunities too by promoting telework practices. Teleworking is one way that workers stay active and engage in their work while they are staying at their homes during the pandemic. Telework is also an attractive and an important option to protect the high‐risk and vulnerable groups during the pandemic and beyond. The increase in teleworking practices during the pandemic could be to the advantage of those who are disproportionately affected by the pandemic such as for women and mothers. Furthermore, the pace of gender norm might be accelerated by the longer hours that fathers are spending with their children. Teleworking could also decrease the inequalities between ethnic minorities (Latino, African American, and Asian). However, still this depends mainly on the characteristics of the jobs, technology availability and training, and productivity.

As economies in many countries are transforming to a knowledge‐based and digital economy, the negative outcomes of pervasive ICT use should not be a concern, such as communication and connectivity. The availability of smart devices (e.g., mobile communication smart devices) allows teleworkers to embrace flexible work schedules. Teleworkers may avoid stress and superfluous communications when they reduce their presence in the workplace; hence, telework practices may provide teleworkers a means to reduce any stressful aspects and filter out some of the distraction in the workplace, making teleworkers' experience greater job satisfaction (Fonner & Roloff, 2010).

## 6 PROMOTING TELEWORK PRACTICES: POSSIBILITIES AND RESPONSIBILITIES

Technology diffusion is essential for successful telework practices but is insufficient to be a successful process on its own. To use technology effectively and efficiently, teleworkers need the correct level of support. Successful telework practices require considering many elements, for example, government policy, management, job autonomy, and land use and digital infrastructure planning. This section discusses these elements in detail.

### 6.1 Government policies

The measures taken by governments worldwide to mitigate the effect of COVID‐19 on human health were effective in many countries, particularly the stay‐at‐home and physical distancing policies. Without these measures, the COVID‐19 virus spread, causing more fatalities and economic and social damage. Telework practices were encouraged by governments to mitigate the negative effects on economic performance at the national and international levels. Organizations encouraged their employees to work from home to avoid losing capital. This shows the importance of the role of government policies in promoting telework practices.

Government policies are significant in a successful telework trend adoption in a virtual society. These policies should aim to support flexible work and to solve problems such as access to digital infrastructure and technologies, access to fast broadband, and digital development; in addition, telework can facilitate other policy goals, for example, reducing the carbon footprint, transport and traffic congestion, and occupational health and safety (Abulibdeh, 2017; Abulibdeh, Andrey, & Melnik, 2015; Hegewisch & Gornick, 2011). The aim of flexible work policies is to help resolve short‐ and long‐term labor shortages, increase labor force participation (i.e., working mothers, protection from discrimination), and address the high unemployment rate in many countries (Hegewisch, 2009).

However, the level of development in various countries is different; hence, the policy priorities may differ between these countries, particularly between underconnected and hyper‐digitalized countries or between developed and developing countries. Many of these countries have many constraints and difficulties that prevent them from benefitting from the knowledge‐based economy or digital economy. Governments should create or support a knowledge‐based economy in their national strategies that should include all major sectors of society, for example, education, health, commerce, civil society, science, and the private sector. This national strategy should develop a knowledge‐based economy in a sustainable, persistent manner. Digital transformation is complex and is evolving rapidly. Therefore, the policy approach toward telework in the context of COVID‐19 mitigation measures and the development of a knowledge‐based economy and digital economy must be coordinated, multidisciplinary, and holistic, and there might be uncertainty about the digital future. Furthermore, these policies require involving relevant stakeholders to enable the design of resilient policy frameworks in response to the pandemic and the evolving digital transformation. The countries should be able to create appropriate mechanisms that enable the collection of required information for producing the intelligence necessary to formulate and implement suitable policies and strategies.

Digitalization can have a crucial positive impact on teleworkers' productivity and the quality of their jobs. Workers and organizations must adapt to technology‐induced changes in the industrial and labor market because of the evolving digital transformation. Teleworkers must learn new digital skills or might lose their jobs as the nature of work changes. Therefore, policymakers and organizations must establish lifelong learning programs to help workers to be better prepared, to adapt, to cope with, and to be more resilient during the transition process, particularly in the virtual world. Furthermore, the increase in the use of digital platforms may result in an increase in work flexibility, which could facilitate telework practices. Finally, public and private sectors in many countries are transforming their services to online services, for example, e‐health, e‐learning, e‐commerce, e‐finance, and e‐banking, which are considered the most innovative, advanced online services and can make telework practices easier in both public and private sectors. Teleworkers may take advantage of e‐services and other digital platforms to enhance their productivity.

Teleworkers can benefit from the transformation of the industry toward a digital era and the investment in various sectors, for example, smart cities, smart retail, smart healthcare, and smart education. Governments can play key roles in promoting telework practices by regulating guidelines and developing policy frameworks for these sectors. Simultaneously, governments can invest in building a technology education infrastructure, capacity building, and innovation solutions. Governments should work with stakeholders and organizations to boost the acceptance and adoption of the telework practices in their transformation to a knowledge‐based economy or digital economy.

On the other hand, countries that have limited capabilities to transform totally or partially to a digital economy and to use digital platforms or turn digital data into business opportunities are at a disadvantage regarding telework practices. These countries should seek to promote telework practices in their national development strategies and in their national digital economic strategy through digital upgrading in their economy and by the collaboration with and support from the international community. Therefore, this necessitates adopting local policies and developing roadmaps to better seize opportunities for digital upgrading and for digitally uplifting society to enhance the probability of adopting telework practices by different organizations while managing the challenges and risks associated with digitizing data, for example, data privacy, control over data, data ownership, and data security.

At the regional and international levels, different policy challenges should be addressed regarding data protection, cross‐border data flows, data security, competition, and trade to organize telework practices when the type of work necessitates the use of international data. Effective international policy dialog and collaboration is significant in finding proper solutions and exploring common policy solutions. As a result of globalization, developing countries should seek more international support to complement their national efforts in the digital transformation process to integrate digital dimensions into their strategies and policies, make telework practices easier, and ensure that these practices contribute to more inclusive outcomes.

### 6.2 Management of telework practices

Management is another significant element in the successful implementation of telework practices. Management of teleworkers is more complex and challenging than managing employees at the workplace, particularly during crises such as COVID‐19 pandemic. Management concerns during an emergency situation are, for example, managerial control, human resource management practices and policies, preventing social and professional isolation, and how to measure employee performance (Blount, 2011). Management incorporates the competencies of managing diverse, complex work arrangements by utilizing technology within a government's policy framework. However, organizational management's capabilities and the managing skills of a complex workforce including teleworking have not been covered in the literature. Therefore, a valuable study would be to investigate the key skills and competencies for managers in managing telework practices and how these skills can be developed. One of these management skills and competencies is the knowledge of the benefits and limitations of telework practices, which allows organizational managers to monitor telework practices to achieve sustainable benefits and mitigate limitations (Van Gramberg, Teicher, & O'Rourke, 2014). Furthermore, good management skills require managers to recognize, understand, and be sensitive to differences in local cultures, particularly in a society of multinational workers. The attitude and behavior of managers toward teleworking are crucial for the adoption of telework practices.

The probability that managers will support telework may increase if managers perceive that they have a high level of control over the work of subordinates and when managers use technology to make decisions in the virtual workplace rather than in a face‐to‐face situation. In the absence of government policy and legislation that support telework, teleworkers must rely on their organizational managers. However, managers may reject the notion of telework if they cannot conceive of the business benefits, and they may by constrained by pressures, for example, work intensification and tough performance targets (Hegewisch, 2009). Managers also have the perception that teleworkers might not focus on their work. Nevertheless, technology can provide managers with media to communicate, collaborate, and monitor teleworkers and customers.

### 6.3 Digital infrastructure planning

Since the prevalence of the pandemic, the pace of adoption of digitalization by enterprises has quickened leading to an increase opportunities for employees to perform their work tasks from home. However, there are variations in the spread of digitalization cross the countries in the globe with some countries, particularly the developing countries, struggling with the lack of advanced ICT, and internet connection, which limit or prevent telework practices. In Sub‐Saharan Africa, for example, only quarter of the population has access to the internet compared to four‐fifth in Europe (Gómez‐Jordana Moya, 2020).

A digital infrastructure, which often refers to a collection of information technologies and systems that jointly produce a desired outcome, is expected to have a transformative impact on telework practices. IoT along with machine‐to‐machine communication comprises fundamental elements of digital infrastructure. This technology allows smart devices to exchange information, collect and share data, create new interactions, and respond to other smart devices. As smart devices become the mainstream in technology, these devices will enable access to a substantial amount of data and the application of real‐time analytics. The array of smart devices used by teleworkers could enhance their performance in unfathomable ways. Teleworkers can benefit from the information generated by connected devices through collecting and analyzing real‐time data, which may provide new business opportunities for their organizations. This also allows teleworkers to satisfy customers during the customer lifecycle. With the availability of digital data, teleworkers working from home could respond to the organization's customers. Many companies are investing in upgrading and digitalizing their systems after considering the value derived from digital infrastructure. Digital infrastructure is the collection of human and technological components, systems, and networks that contribute to the functioning of an information system (Braa, Hanseth, Heywood, Mohammed, & Shaw, 2007; Tilson, Lyytinen, & Sorensen, 2010) and can transform how information is created, stored, transmitted, and used. The evolution process of the digital infrastructure encompasses both social and technical factors and is the process where autonomous and heterogeneous organizational, or human, actors tend to use IT in their adaptation to their external environments and each other (Braa et al., 2007; Hanseth, Jacucci, Grisot, & Aanestad, 2006; Vaast & Walsham, 2009).

Investments in digital infrastructure (i.e., IoT, 5G, new fiber‐to‐the‐premises, new broadband strategy) to facilitate new methods of communicating can facilitate telework practices. Many vendors have introduced gateways to increase the connectivity between new devices to local computer platforms. Digital infrastructure has levels: ICT networks, data infrastructure, digital platforms, digital devices, and applications. ICT networks are considered the core digital infrastructure of connectivity. To make ICT networks more effective, network capabilities should be expanded by network providers to make their networks compatible with connected devices. The progress in several frontier technologies is one of the major components that lead to the evolution of telework practices. These technologies include some key software‐oriented technologies, such as AI, blockchain, IoT, cloud computing, and data analytics. Rapid advances in these technologies are an advantage for telework practices because teleworkers may benefit from the increased capacity in telecommunication networks and the cost reduction in communication and data storage and processing. Furthermore, teleworkers may use or develop many applications to bring in new services and features from the digital infrastructure to enhance their work‐from‐home practices and connect to their workplace and the customers. Digital applications are significant for the efficient functioning of connected devices, particularly in real‐time data exchange. Applications possess important milestones for teleworkers for running their work smartly.

Digital infrastructure can facilitate telework; hence, government policy associated with investments in digital infrastructure should provide opportunities for employees to telework. With the emergence of the IoT, computing power, communication network speed, and data storage, many organizations in different countries are transforming to digitalization, which require strong digital infrastructure. IoT is a crucial parameter of digital infrastructure and a new paradigm that facilitates the increased performance and coordination of existing technologies, which teleworkers can use to perform their work that fulfills high‐quality standards. However, if not controlled appropriately, the IoT could be misused, as could occur for any entity with such power, and create dangerous outcomes. Therefore, security, privacy, and ownership of data remain major issues and big challenges and must be managed holistically. Furthermore, technology support is required by the organizations as it is significant to the success of telework practices; therefore, fixing IT problems quickly and efficiently is crucial for teleworkers' productivity.

### 6.4 Job autonomy

Job autonomy is an essential feature for the successful implementation and adaptation of telework practices in different organizations. Job autonomy allows teleworkers to make decisions on when, how, and where to work to achieve their duties and to attain the organizational objectives, through having an explicit agreement with organizational management on expected outcomes (productivity). Job autonomy results in a greater level of job satisfaction for teleworkers. In addition, job autonomy is associated with improved work well‐being, for example, psychological flexibility, successful telework, self‐realization, and improved vitality (Lopes, Lagoa, & Calapez, 2014). To promote job autonomy in telework practice, teleworkers should have the potential for self‐development and a great sense of achievement and efficiency, just as they would when working in a regular office.

In the era of the digital economy, digital data can increase the level of job autonomy because control of data by teleworkers enables them to transform the data into digital intelligence. Organizations and teleworkers can use these digital data as the core for all fast‐emerging digital technology, such as IoT, cloud computing, AI, data analytics, and all internet‐based services. Currently, data‐centric business models are being adopted by lead companies across different sectors and by digital platforms, which enable teleworkers to work with confidence.

## 7 CHALLENGES FACING THE IMPLEMENTATION OF TELEWORK PRACTICES

Despite the advances in technology and the transformation to a knowledge‐based and digital economy, teleworkers, organizations, and governments will probably have to overcome distinct challenges before telework is successful. Teleworkers with no or limited digital skills will be unable to perform the work effectively and will be at a disadvantage vis‐à‐vis those who have good knowledge and are better equipped for a knowledge‐based or digital economy. On the organizational level, competition may occur on two levels: first, between national companies, because compared with nondigitized companies, digitized companies will have the lead in the market compared with other nondigitized companies; and second, between digitized foreign companies and digitized domestic companies. Hence, the net impact in these cases depends on the level of development, policies adopted at different geographical scales, and digital readiness of countries.

Employees working in organizations and companies without any previous teleworking practices may encounter a lack of clarity around the tasks and priorities they need to accomplish. Other challenges may face those new teleworkers include the uncertainty about where to get specific support, whom to talk to on specific issues, and when and how to approach colleagues. Furthermore, employees have to get use and familiarize themselves with different or new technological tools in addition to switching to a different way of organizing of work. This might lead to holdups and delays and explains partially why many workers perform their work tasks in a longer time (Bick et al., 2020).

Accordingly, telework practices can be in their initial or advanced stages because these practices mainly depend on the emerging technology and the availability and affordability of ICT access, because it helps teleworkers to become more productive and able to achieve the organizational objectives. In contemporary organizations, the pervasive adoption and use of ICTs make the relationship between information systems and organization increasingly complex (Grundke et al., 2018; Zammuto, Griffith, Majchrzak, Dougherty, & Faraj, 2007). Many organizations face the challenges of controlling an array of systems and technologies as information systems become interconnected. Furthermore, organizations must provide the appropriate technology support for teleworkers to perform their duties. Data security and data privacy, particularly the data transfer between devices, are other risks and challenges for organizations. Governments and organizations must focus on these risks and invest in high‐end security arrangements to prevent these risks. Governments must regulate the counter theft of personal data to ensure that no breach of personal data by teleworkers occurs and that data‐driven business models generate gains for society as a whole. Solving these challenges will provide an organization with a competitive advantage. On the country level, the main challenges for countries is their capability to continually invest in state‐of‐the‐art technologies and their capabilities to drive their digital development to overcome the challenges that manifest adopting and implementing policies that promote telework practices. Furthermore, governments must support and boost entrepreneurship in the digital and digitally enabled sectors so that they become effective; hence, telework practices can be more acceptable to these organizations. Furthermore, the low level of e‐services in developing countries is a barrier and a challenge to scaling the activities of the organizations in the digital economy.

## 8 CONCLUSION

Prior to the pandemic, telework practices varied substantially across countries, occupations, sectors, and firms. However, during the pandemic, telework practices increased substantially and telework practices may remain essential and permanent feature of the future of many private enterprises and public sector benefiting from the experience gained from practicing teleworking during the pandemic. In the long run, the widespread of telework practices during the pandemic has the potential to maintain economic activities as well to enhance and improve productivity and other social and economic indicators such as gender equality, worker well‐being, and regional inequalities. To benefit from this experience, policy makers have to work to improve the gains from more widespread teleworking for innovation and productivity by promoting the diffusion of self‐management, ICT skills managerial best practices, fast and reliable broadband, and investments in home offices across the country.

COVID‐19 has affected society and the economy. People have had to stay at home, and many organizations were closed to prevent the spread of the disease. The resulting increase in telework practices makes how these practices can be encouraged by individuals, organizations, governments, and society a valuable topic of research. Telework practices at the time of risk are significant in reducing economic loss. The transformation toward a knowledge‐based and digital economy in many countries promotes telework practices and can add value to them. Transforming to a knowledge‐based or digital economy requires the collaboration of the international community to form policies to help developing countries increase their capabilities to involve more users and consumers in these economies; only then can the process of digitalization fully support telework practices on the national and international levels. However, the challenge is enormous because this process requires capabilities, particularly in developing countries, and requires involving the adoption and adaptation of policies, regulations, and laws in many areas including the digitalization of many sectors and telework practices. However, many sectors cannot adopt telework practices because of the nature of this work. Therefore, telework practices depend on the extent to which the designing of all jobs to perform by using telework practices is possible, desirable, or realistic and has significant implications for the success of telework.
